# The Effect of the Restrictive Ketogenic Diet on the Body Composition, Haematological and Biochemical Parameters, Oxidative Stress and Advanced Glycation End-Products in Young Wistar Rats with Diet-Induced Obesity

**DOI:** 10.3390/nu14224805

**Published:** 2022-11-13

**Authors:** Natalia Drabińska, Jerzy Juśkiewicz, Wiesław Wiczkowski

**Affiliations:** 1 Department of Chemistry and Biodynamics of Food, Institute of Animal Reproduction and Food Research, Polish Academy of Sciences, Tuwima 10 Str., 10-748 Olsztyn, Poland; 2 Department of Biological Functions of Food, Institute of Animal Reproduction and Food Research, Polish Academy of Sciences, Tuwima 10 Str., 10-748 Olsztyn, Poland

**Keywords:** ketogenic diet, obesity, oxidative stress, advanced glycation end-products, low carbohydrate diet

## Abstract

Over the past few years, the interest in the application of the ketogenic diet (KD) for obesity management is growing. Although many studies have been performed on the effects of KD, the metabolic and physiological impact of KD is still not fully understood. Therefore, this study aimed to evaluate the effect of calorie-restricted KD on the body weight and composition, oxidative stress, and advanced glycation end products (AGEs) assessed in an animal model with young Wistar rats. KD was followed for 4 weeks in maturity after an obesity-inducing high-fat diet during adolescence, resulting in a slowing down of the weight gain but higher adiposity compared to a standard diet. Increased adiposity resulted in an deterioration of liver parameters, suggesting negative changes in this organ. No adverse effects of KD were determined in haematological parameters in young rats. KD did not affect AGEs; however, a decrease in oxidative stress was observed. Based on the presented results, it can be concluded that KD applied for weight loss in obesity induced in adolescence may reduce oxidative stress without compromising the haematological status; however, caution may be required to control adiposity, glucose level and liver health. Thus, KD therapy should be carefully controlled, especially in young subjects.

## 1. Introduction

In recent decades, obesity reached an epidemic scale and became a serious problem for public health. Obesity can lead to chronic health consequences; therefore, there is an urgent need to search for its treatments. Common comorbidities of obesity are type 2 diabetes, cardiovascular diseases and increased risk for cancer development [1]. The severity of obesity-related diseases is closely related to body fat distribution, and in particular to visceral localisation. Visceral fat accumulation is linked to the status of adipokines, consequently inducing chronic inflammation and metabolic disorders [2]. Primary adipokines secreted by visceral adipose tissue play a role in inflammation, metabolism and production of reactive oxygen species (ROS), leading to oxidative stress. Several mechanisms are involved in enhancing oxidative stress, including the generation of free radicals by activated immune cells [3].

Overeating highly processed food can lead to an increased level of cytotoxic, prooxidant compounds known as advanced glycation end-products (AGEs) [4]. AGEs are formed in a nonenzymatic Maillard reaction between reactive sugars and proteins; therefore, hyperglycaemia is suggested as the main source of endogenous AGEs [5,6]. The studies showed that increased circulating AGEs have proinflammatory effects and are associated with insulin resistance and the development of metabolic syndrome independent of energy balance [4,7]. Through binding to their receptor, AGEs initiate the generation of ROS and proinflammatory signalling cascade, which lead to the development of inflammatory diseases [8]. Since sugars are the main substrate in the Maillard reaction, the limitation of carbohydrate consumption may be assumed to reduce the formation of endogenous AGEs.

The ketogenic diet (KD) is a dietetic regime with a limited intake of carbohydrates, which consists mainly of the consumption of fat. In a classical KD, around 80–90% of calories are derived from fat [9,10,11]. Among other low-carbohydrate diets, the KD is distinguished by the presence of a ketosis state. During ketosis, the shift from glucose to fatty acids as the main respiratory substrate leads to the increased production of ketone bodies (acetone, acetoacetate, and β-hydroxybutyrate) [12]. Although ketosis is mainly associated with diabetes, physiological, low-intensity ketosis is observed every day after overnight fasting. For comparison, after overnight fasting, the serum ketone concentration is around 0.06–0.09 nmol/mL, while diabetic ketoacidosis can reach even 25 nmol/L [13]. In individuals following the KD for weight loss, the serum ketone level was estimated at 0.33–0.72 nmol/L [14]. Originally, the KD was developed for the treatment of epilepsy, especially drug-resistant seizures [11]. However, over the past few years, the interest in the application of KD for obesity management is growing [9]. The KD was found to reduce body weight, body mass index (BMI) and fat mass; however, it is still not clear if it was the effect of the KD or calorie restriction, since the majority of studies were performed with the very-low-calorie KD, which consists of just 500–800 kcal per day [15,16]. The clinical trials showed that KD can positively affect the level of circulating interleukin (IL) 6 and 8, metalloproteinase 2, C-reactive protein, tumour necrosis factor-alpha (TNF-α) and resistin as well as lipid metabolism [17,18,19,20]. Many of the studies so far did not contain the control groups and it cannot be confirmed whether the changes were caused by the KD or were a result of the body weight reduction. Contrarily, some of the studies reported negative effects of low-carbohydrate diets including impaired insulin response and atherosclerosis [21,22]. There is no consensus about the effect of KD on oxidative stress, which in some studies was improved [23] and in others elevated [24]. The effect of KD on serum AGEs has not been studied yet, however, the hypothesis that KD can enhance AGEs formation was recently proposed [25]. The mechanism underlying the effectiveness of KD for obesity management is still unknown although it is suggested that altered appetite regulation, reduced availability of foods or loss of total body water, especially in the initial phase are the most important factors [26].

An important consideration is the effect of KD in children and adolescents, for which KD is applied for the treatment of intractable epilepsy. It has been reported that KD is affecting normal longitudinal growth, especially in young children, although was not applied as a restrictive diet [27]. The putative reason for that is the limitation of growth hormones and insufficient lean body mass [26]. Individuals with a growth hormone deficiency demonstrate an altered body composition with increased fat mass, especially visceral fat [28]. Increased fat mass after KD incorporation was reported previously in rats with obesity [29]. Therefore, changes in body composition can be expected after consuming KD for either epilepsy or weight loss. The prevalence of obesity in children has been dramatically increasing worldwide over the past decades, becoming a major global challenge. Obesity in childhood tends to persist into adulthood and is an early risk factor for obesity-associated morbidity and mortality, highlighting the importance of early intervention [30]. Therefore, studies on the effect of dietary treatments in adolescents on health-related parameters and their consequences in adult life are needed.

Since the effect of the KD is not conclusive, more studies are needed to understand the physiological effects of the KD. Therefore, this study was designed to evaluate the effect of calorie-restricted KD on the body weight and composition, biochemical and haematological parameters, oxidative stress and AGEs assessed in an animal model with young Wistar rats which had diet-induced obesity in adolescence. In this study, an extreme-in-composition KD was applied (>90% of energy from fat) after adolescence to examine the effects of severe carbohydrate restriction while at the same time limiting gluconeogenesis from dietary protein. Considering the effects of KD on the body composition in children, irrespectively of the reason for the applied diet, the current investigation was conducted on adolescent rats with diet-induced obesity to further explore the biochemical response to KD in adults, which was less elucidated to date.

## 2. Materials and Methods

### 2.1. Animals and Diets

The in vivo schema and all manipulations performed on living rodents followed the rules of the European Union Directive (2010/63/EU) for animal studies, and the experiment was conducted with the Local Institutional Animal Care and Use Committee’s permission (No. 34/2019; Olsztyn, Poland). The study was also carried out in compliance with the ARRIVE guidelines. Every effort was made to minimize the suffering of the animals used in the experiment.

**Figure 1:**
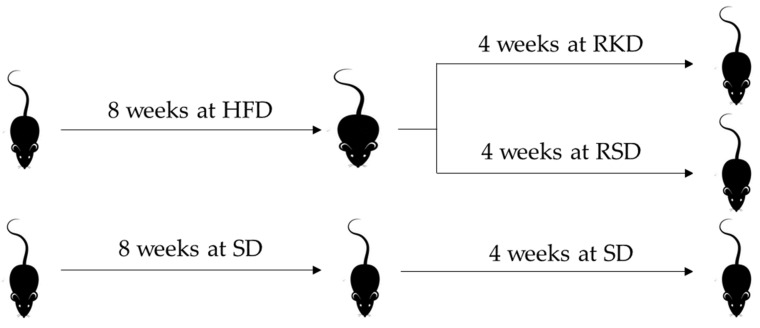
The design of the animal study. HFD—high-fat diet; SD—standard diet; RKD—restrictive ketogenic diet; RSD—restrictive standard diet.

The nutritional intervention was conducted on 34 male Wistar rats (*Rattus norvegicus* Cmdb:WI), which were allocated to three experimental groups housed individually in balance cages under a constant temperature (22 ± 1 °C) and a 12/12 h light/dark cycle. Group S (n = 12) was fed a standard diet (SD) containing 8% soy oil as a fat source and 8% cellulose as a source of dietary fibre for the whole experiment (initial and experimental periods). Rats in groups RKD and RSD were initially fed a high-fat diet (HFD) for 8 weeks, where the diet contained 23% lard (at the expense of corn starch). After 8 weeks, animals were randomly assigned into RKD and RSD groups, which were fed with KD, and a standard diet with 20% caloric restriction compared to SD, respectively. The RKD group was fed for 4 weeks with TD.96355 Ketogenic diet (Harlan Laboratories, Madison, WI, USA), which consisted of 91.6% of calories from fat, 8.4% calories from protein and 0% calories from carbohydrates. The design of the study is presented in Figure 1.

The planned number of animals was set with the size of one experimental group n = 6, which is the minimum number of laboratory animals with a uniform genotype, which ensures obtaining reliable, reproducible and statistically significant results without the need to repeat the procedure due to high intra-group variability. Taking into account the adopted methodology, the desired test power should be 80% (taking into account the most important parameters that were analysed in the blood and parameters from the NMR test). Statistical algorithms developed by Columbia University Medical Center (www.biomath.info) were used. Based on the variables (predicted parameter values with the highest predicted variability based on previous own research, i.e., NMR and redox status) and online software, it was calculated, assuming the use of a parametric test, that the number of animals in the group should be just 6 [31].

After the initial period (standard and high-fat diets) and after each week of feeding regimens with reduction or standard diets, animals were subjected to time-domain nuclear magnetic resonance (NMR) using the minispec LF90II Body Composition Analyzer (Bruker. Karlsruhe. Germany) to determine fat and lean body mass.

### 2.2. Sample Collection

Before the reduction diets (6 rats each of SD group and restrictive groups) and at the end of the experiment, the rats fasted for 12 h and were anaesthetized *i.p.* with ketamine (K) and xylazine (X) (K, 100/kg BW; X, 10 mg/kg BW) according to recommendations for anaesthesia and euthanasia of experimental animals. Following laparotomy, blood samples were taken from the caudal vena cava into heparinized tubes, and finally, the rats were euthanized by cervical dislocation. The blood plasma was prepared by solidification and low-speed centrifugation (350× *g*, 10 min, 4 °C). Plasma samples were kept frozen at −70 °C until being assayed.

### 2.3. Biochemical and Haematological Analyses

The following haematological parameters were determined in whole heparinized blood using the ABACUS Jr VET Analyzer (DIATRON MI PLC, Budapest, Hungary): WBC—total white blood cell; LYM—lymphocyte; MID—medium-sized cell; GRA—granulocytes; RBC—red blood cell; HGB—haemoglobin; HCT—haematocrit; MCV—mean corpuscular volume; MCH—mean corpuscular haemoglobin; MCHC—mean corpuscular haemoglobin concentration; RDWc—red cell distribution width; PLT—platelet count; PCT—platelet percentage; MPV—mean platelet volume; PDWc—platelet distribution width.

The level of glucose, total cholesterol, HDL cholesterol, triglycerides (Tg), uric acid, urea, creatinine and bilirubin, as well as activity of alanine aminotransferase (ALT), aspartate aminotransferase (AST), alkaline phosphatase (ALP) and gamma-glutamyl transferase (GGT) were measured using an automatic biochemical analyser (Pentra C200, Horiba, Kyoto, Japan). The atherogenic index of plasma (AIP) was calculated as the log value of the ratio between Tg and HDL.

The leptin plasma concentration was analysed using a commercial ELISA kit, code: EM0129 (FineTest, Wuhan, China). The detection range of the test was 62.5–4000 pg/mL, the sensitivity was <37.5 pg/mL, and the coefficient of variation was below 10%.

The ketone body content was estimated using Keto Diasticks^TM^ (Bayer, Leverkusen, Germany).

### 2.4. Oxidative Stress Markers

Malonaldehyde was analysed using a commercial ELISA kit, code: EM1723-1 (FineTest, Wuhan, China). The detection range of the test was 7.813–500 ng/mL, the sensitivity was 4.688 ng/mL, and the coefficient of variation was below 10%.

Superoxide dismutase (SOD) was analysed using a commercial ELISA kit, code: EM0419 (FineTest, Wuhan, China). The detection range of the test was 0.781–50 ng/mL, the sensitivity was 0.469 ng/mL, and the coefficient of variation was below 10%.

AGEs were analysed using an ELISA kit ER0268 (FineTest, Wuhan, China). The detection range of the test was 0.313–20 ng/mL, the sensitivity was 0.188 ng/mL, and the coefficient of variation was below 10%.

### 2.5. Statistical Analyses

All the analyses were conducted in triplicates. The differences between the study groups were compared using one-way ANOVA with Fisher’s LSD test as a post hoc or using the Kruskal–Wallis test, depending on normality evaluated by the Shapiro–Wilk test. The results are presented as mean ± standard deviation or by median (Q1; Q3), depending on normality. The differences with a *p*-value < 0.05 were considered significant. Differences in body composition were compared using two-way ANOVA to assess the effect of time (T) and type of diet (D), and interactions thereof (T × D). Correlations between parameters were analysed using Spearman’s rank correlation coefficient test, separately for each group and each study interval. All the statistical analyses were performed using STATISTICA version 13.3 (TIBCO Software Inc., Palo Alto, CA, USA) software.

## 3. Results

### 3.1. The Effect of the Ketogenic Diet on Body Weight and Composition

**Figure 2:**
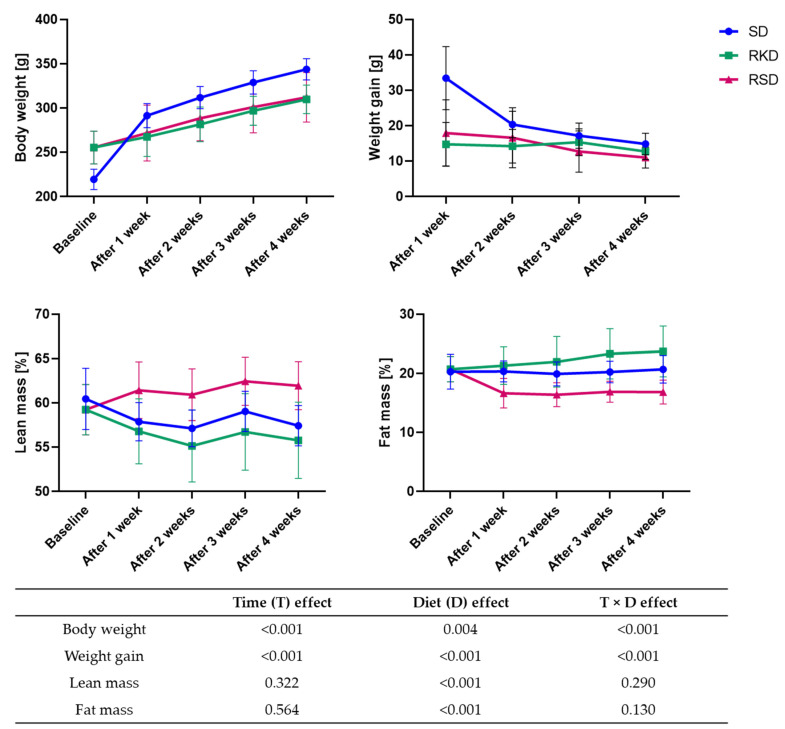
The changes in the body weight, weight gain, percentage of lean and fat mass during 4 weeks of KD or control diet. The *p*-values of the two-way ANOVA analysis are presented in Table.

The effect of KD and SD on body weight, weight gain and the percentage of lean and fat mass is presented in Figure 2. The rats from the SD group, which were fed the same diet for the duration of the experiment, had significantly lower body weight at the beginning of the study. However, their body weight gradually increased with time. The biggest gain in weight was observed after 1 week of the diet. Rats from the RSD and RKD groups also gained weight, but much more slowly than the SD group. The weight gain was initially slightly higher in the RSD than the RKD group; however, after 3 weeks, this trend was the opposite. The lean mass was initially slightly higher in the SD group compared to the rats with obesity-inducing HFD. However, when the reduction was initiated, the percentage of lean mass was increased in RSD, while for RKD, a tendency similar to the SD group was observed. The HFD prior to the reduction had no effect on the fat mass. A stable level of fat mass was observed for the SD group, while for restrictive groups the opposite changes were noted. The fat percentage in RKD increased with the prolongation of KD, while the standard diet incorporated after HFD in the RSD group resulted in the reduction of fat mass. Interestingly, the results of the two-way ANOVA showed that type of diet and time had a significant effect on body weight and weight gain, while time had no significant effect on lean and fat mass. The significant interaction between time and type of diet was noted for all analysed parameters.

### 3.2. The Effect of the Ketogenic Diet on Blood Haematology

The results of the haematological parameters are presented in Table 1. The initial HFD resulted in significant changes in blood haematology. After HDF, lower HGB and MCH levels were observed. Moreover, lower MID, both in concentration and in percentage were noted. Finally, HFD resulted in an elevated level of PLT.

After the next 4 weeks of growth, higher RBC and PDWc with simultaneous lower MCH were noted for rats in the SD group. In the RKD group, the highest RDWc and HCT were observed. In comparison to SD, the restrictive diets after HFD resulted in lower white blood cell system parameters, as well as MPV and PDWc, irrespectively of the type of diet. Comparing the two restrictive diets, significantly higher HBG and HCT were noted for RKD compared to RSD.

### 3.3. The Effect of KD on the Biochemical Parameters

The level of ketone bodies measured in urine appeared to be elevated in the RKD group from the second week till the end of the restriction. The results of parameters associated with lipid profile, liver and kidney functioning are presented in Table 2. The lipid profile was affected by the initial HFD-altering triglycerides, HDL, LDL and AIP. In the SD group, no changes were observed in the lipid profile at the end of the experiment. The rats from the RSD group had the lowest level of cholesterol, triglycerides, HDL and AIP after 4 weeks of reduction. In the RKD diet, the highest level of HDL was noted at the end of the experiment. LDL level normalized in all analysed groups at the end of the experiment. AIP level in RKD was the highest among all groups at the end of the experiment; however, it was significantly reduced compared to the HFD group. Interestingly, the glucose level was the highest in both restrictive diets.

Liver parameters, namely AST and ALT, were significantly elevated after initial HFD. In the SD diet at the end of the experiment, a significant reduction in ALT and ALP was observed. After 4 weeks of a restrictive diet, the level of AST and ALP was the highest in the RKD group, and the level of ALT was the highest among the rats at the end of the experiment. None of the groups reported an elevated level of GGT or bilirubin. 

Finally, the kidney parameters, namely uric acid and urea, were elevated in the rats on initial HFD. After 4 weeks, in the RKD group, the lowest level of these parameters was observed. The highest level of creatinine was reported in the RSD group.

**Figure 3:**
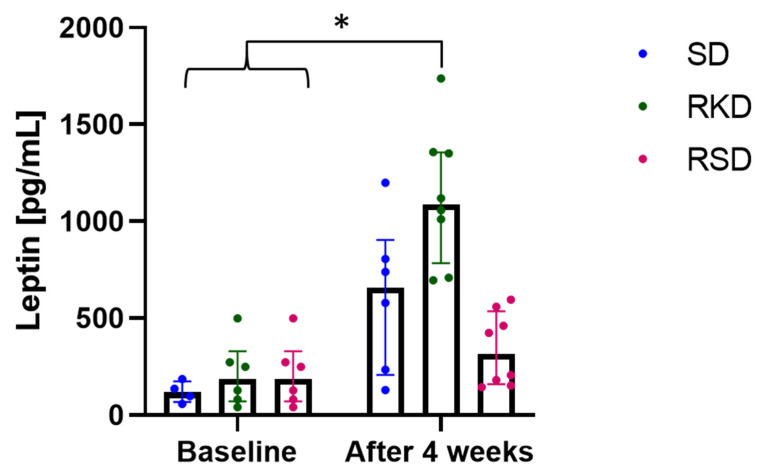
The status of leptin before and after the nutritional intervention. The bars represents the median with the error bars representing interquartile range (Q1; Q3). The significant differences are marker with “*”.

The plasma concentration of leptin was slightly higher in groups after initial HFD than in the SD group at the baseline (Figure 3). After the restrictive diet, a significantly higher level of this hormone was observed in the RKD group, while the lowest concentration was observed in RSD. In the SD group, leptin level increased 5 times after 4 weeks of growth.

### 3.4. The Effect of the Ketogenic Diet on the Oxidative Status

**Figure 4:**
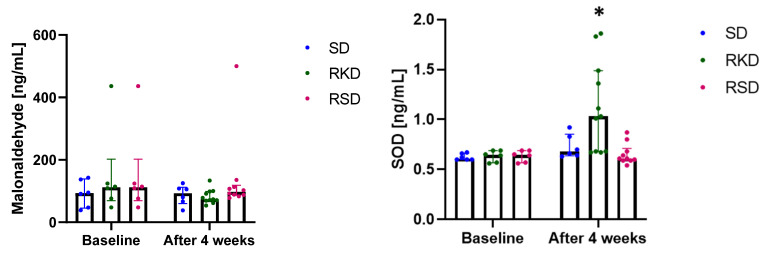
The status of malonaldehyde and SOD before and after the nutritional intervention. The bars represents the median with the error bars representing interquartile range (Q1; Q3). The significant differences are marked with “*”.

In this study, the plasma concentration of malonaldehyde and SOD were detected to evaluate the oxidative stress and the results are presented in Figure 4. Although no significant differences were observed for malonaldehyde (Figure 4), clear tendencies could be noticed. At the baseline, a much lower content of malonaldehyde was observed for the SD group compared to rats on an HFD. After 4 weeks, the level of malonaldehyde remained at a similar level in the SD group. After restrictive diets, in RKD the malonaldehyde level decreased compared to baseline and was similar to the SD group. At the same time, in the RSD group, the plasma concentration of this marker remained similar to the baseline in HFD group.

Much more pronounced changes were noted for SOD (Figure 4). At baseline, no changes were observed between the studied groups. In the SD and RSD groups, the level of this marker remained unchanged. However, in the RKD group, a significant increase in SOD was observed.

**Figure 5:**
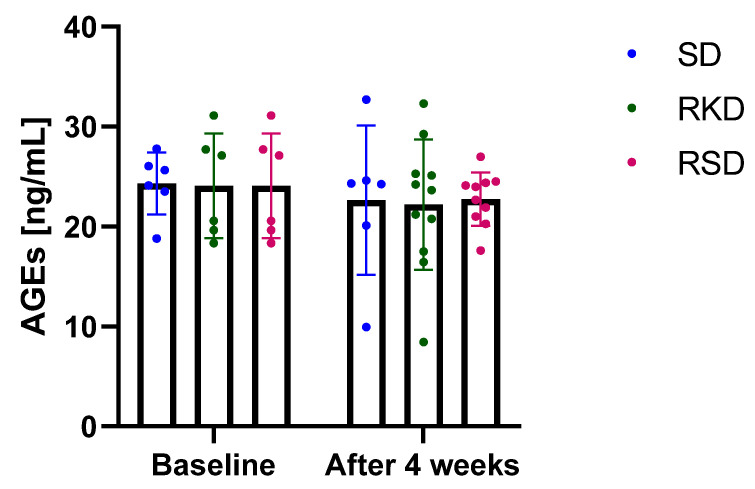
The plasma concentration of advanced glycation end-products (AGEs) before and after the intervention. The bars represents the mean with the error bars representing standard deviation.

The results regarding AGEs are presented in Figure 5. Neither the initial HFD nor the restrictive diet afterwards influenced AGEs. A small decrease in AGEs was noted after 4 weeks but was similar for all study groups.

### 3.5. Correlations between Body Composition and Biochemical Parameters

The results of the correlation analysis in all studied groups before and after the intervention are presented in Figures S1–S5. In the SD group at the baseline, significant positive correlations were detected between body weight and MDA (r = 0.886) and SOD (r = 0.829); ALP and HDL (r = 0.841); and cholesterol and HDL (r = 0.812). The negative correlations in the SD group were found between fat mass and lean mass (r = −0.943); ALT and leptin (r = −0.900); and urea and Tg (r = −0.886).

In the group fed with HFD at the baseline, positive significant correlations were observed between body weight and fat mass (r = 0.886); cholesterol and glucose (r = 0.829) and AGEs (r = 0.943); Tg and AIP (r = 0.829); and glucose and AGEs (r = 0.943). In this group, negative significant correlations were detected between body weight and lean mass (r = −0.886); ALT and creatinine (r = −0.829) and cholesterol (r = −0.829); and HDL and AIP (r = −0.928).

After the intervention, in the group fed with the SD diet for the duration of the experiment, positive significant correlations were detected between body weight and leptin (r = 0.943); fat mass and leptin (r = 0.943); ALP and creatinine (r = 0.829); Tg and AIP (r = 0.943); and glucose and AGEs (r = 0.943). At the same time, negative significant correlations were observed between fat mass and lean mass (r = −0.943); ALT and glucose (r = −0.829); and cholesterol and glucose (r = −0.886).

In the RKD group, positive, significant correlations were detected between fat mass and SOD (r = 0.786); lean mass and ALT (r = 0.714) and ALP (r = 0.762); AST and ALT (r = 0.855); ALT and ALP (r = 0.655); uric acid and MDA (r = 0.714); urea and Tg (r = 0.627); cholesterol and AGEs (r = 0.779); and Tg and AIP (r = 0.973). Negative significant correlations in the RKD group were detected between: fat mass and ALT (r = −0.714) and ALP (r = −0.762); lean mass and SOD (r = −0.786); Tg and MDA (r = −0.782); AIP and MDA (r = −0.800); and SOD and AGEs (r = −0.636).

In the RSD group, positive significant correlations were detected between body weight and urea (r = 0.762); lean mass and HDL (r = 0.790); ALT and ALP (r = 0.661); Tg and AIP (r = 0.927); and SOD and AGEs (r = 0.842). In the RSD group, negative significant correlations were noted between body weight and ALT (r = −0.810) and HDL (r = −0.743); fat mass and lean mass (r = −0.976) and HDL (r = −0.838); ALT and urea (r = −0.855); ALP and cholesterol (r = −0.687); urea and HDL (r = −0.718); Tg and MDA (r = −0.661); and AIP and MDA (r = −0.685).

## 4. Discussion

In the current investigations, we were able to confirm and extend the existing knowledge on the effects of a restrictive KD on body weight and composition as well as haematological and biochemical parameters, in addition to highlighting the effect of a restrictive KD on oxidative stress.

In the young Wistar rats, the restrictive KD resulted in a slowdown in the gain of body weight, similar to the restrictive SD. This suggests that caloric intake seems to have more importance than diet type. However, the body composition differed significantly between the analysed restrictions. Rats fed with KD had more fat mass and less lean mass compared to the RSD group. This is in agreement with previous reports, which also observed an increase in body fat percentage after KD intake [29,32,33]. It is probably a consequence of the composition of KD, leading to the deposition of fat in various tissues, including the liver, which in turn can lead to the development of non-alcoholic fatty liver disease (NAFLD) [34]. The lower lean mass in rats fed with KD observed in this study can be explained by lower protein intake, which is required to maintain muscle growth, especially in young organisms. The concerning fact is that the reduction of body weight in rats from the RKD group resulted mainly from losing the lean mass due to the lower protein intake. The appropriate amount of muscle is of particular importance in the young, still-growing organisms. Therefore, this must be kept in mind before the introduction of KD. Another explanation can be related to the deficiency in the growth hormone as reported by Caton et al. [26]. However, the growth hormone was not analysed in this study and therefore cannot be confirmed. Further studies on this topic are required. A limited number of reports focused on the effect of KD in immature animals with obesity. In this case, a decrease in body weight is not expected after introducing the restrictive diet, since the animals are still growing. In the study of Caton et al. [26], who examined the effect of KD in non-obese, young rats, the lack of body weight gain was reported, contrary to the reduction in body mass in adult rats. This is in accordance with this investigation, since in our study, rats with HFD-induced obesity were analysed, and the differences between the rats from the RKD and SD groups were significant. The cited authors underlined that the initial body weight has a crucial impact on the response to KD [26].

In this study, an elevated concentration of leptin was observed in rats fed with a KD. Leptin is considered an indicator of higher fat mass [35], which is consistent with the findings of the body composition determined in this study. However, the significant positive correlations between leptin and fat mass were observed only in the SD group after the intervention. Leptin is controlled by the hypothalamus and is focused to reduce food intake and limit adiposity [36]. In the animals with HFD and non-KD-induced obesity, the anorectic effect of leptin was suppressed [37]. However, the study of Kinzig et al. [35] showed that although the fat mass percentage was elevated in the rats fed with KD, the loss of sensitivity to leptin was not observed like in HFD. Moreover, the level of peripheral leptin was reported to be dependent on the triglyceride level [38]. Triglycerides inhibit the transport of leptin through the blood–brain barrier, participating indirectly in the resistance to leptin. In our study, although rats from the RKD group had an extremely high level of dietary fat, the concentration of triglycerides was not higher compared to other groups. Additionally, we did not observe the correlation between the level of leptin and Tg in any of the groups. It may be speculated, taking into account the lack of triglyceride elevation with the known lack of loss in sensitivity to leptin despite high adiposity after KD intake, that a following KD in obesity can preserve the beneficial effects of leptin, supporting the limitation of the food intake and increased satiety. However, this requires a confirmation in the experimental studies. A similar observation regarding the lack of triglycerides increasing after KD was reported by Kinzig et al. [35].

The positive effect of KD on the lipid profile was repeatedly reported in clinical trials [9,39,40]. For instance, only two weeks of low-calorie KD were enough to reduce the cholesterol, triglycerides and LDL cholesterol in individuals with obesity [39]. In the current investigation, KD did not have a substantial effect on the lipid profile. Notably, the level of HDL cholesterol was elevated after KD intake. Contrary to other investigations [41,42] which reported a decrease in glucose level after KD, in this study, elevated glucose concentration was noted for both restrictive groups. It is difficult to explain, especially in rats fed with KD where the carbohydrate level was extremely low, why the glucose level increased. The possible explanation of this phenomenon could be associated with potential inhibitory effect of insulin on gluconeogenesis in the liver due to insulin resistance. This could explain the increased glucose level in rats from the RKD group. Another possible explanation is related to the fact that, since KD resulted in the decrease in the lean mass and consequently less muscles to take up glucose, this could lead to a higher level of “unconsumed” glucose in the bloodstream. This issue requires further elucidation in future. In this investigation, AIP was calculated to estimate the risk of the development of cardiovascular diseases. AIP was found to be a useful marker of coronary and metabolic syndrome [43]. In rats fed with HFD, AIP doubled compared to the rats fed with SD. For humans, it is considered that AIP > 0.21 represents a high risk of atherosclerosis [44]. However, this range was never adjusted to the animal model. In our study, AIP = 0.21 was reported in young, healthy rats fed with SD, for which the elevated risk of cardiovascular diseases is not expected. The restrictive KD, similarly to RSD, resulted in a decrease in this parameter. This suggests that only the calorie restriction, not the type of diet, has a stronger effect on the reduction of the development of cardiovascular diseases. At the end of the experiment, in rats fed with KD, the optimal AIP value was slightly exceeded, which would suggest a higher risk of atherosclerosis. However, considering that in the SD group before the intervention, the AIP was 0.21, then the increased risk of coronary problems caused by KD (AIP = 0.23) was not expected. In our study, AIP was positively correlated with Tg in all analysed groups and negatively correlated with HDL in the group with initial HFD and with MDA in the RKD and RSD groups.

Obesity can lead to the development of liver impairment, being the consequence of the accumulation of lipids in the liver [34]. In the current study, the initial obesity-inducing HFD resulted in an elevated level of the activity of liver enzymes. The restrictive KD, despite the inhibiting effect on the body weight similar to RSD, resulted in a maintaining of a high level of all liver-related parameters. This is in agreement with previous studies, which showed that KD intake can induce hepatic steatosis in mice [45]. Moreover, the KD was found to induce liver changes, which could potentially result in insulin resistance despite the prevention of body weight gain and increased energy expenditure [46]. The elevated level of liver enzymes, which may suggest the development of NAFLD in future, may have an important clinical implication in carefully selecting KD for obesity treatment, especially in young organisms.

Long-term adherence to KD has been suggested as a risk factor for the development of kidney stones and increased level of circulating uric acid [47,48]. Since KD as a therapy for obesity is usually followed for a few weeks, the negative effect of this diet on the kidneys has not been confirmed in clinical trials with individuals with obesity, even with mild kidney failure [49]. In spontaneously hypertensive rats, the reduction in renal autophagy levels and elevated renal parenchymal damage were reported after a 4-week KD intervention [50]. The authors detected elevated creatinine levels in the rats fed with KD. In the current study, no negative effects on kidney-associated parameters were reported. The creatinine level in RKD was higher than at the baseline; however, it was at the same level as in rats fed with SD for the whole experiment, thus the increase in creatinine could be a result of the growth of rats. Only in rats from the RSD group was elevated creatinine noted. The discrepancies between our study and other trials with rodents, which reported an increase in creatinine [50,51], can be associated with age, since in these studies the adult rats were analysed, while in ours the adolescent rats with diet-induced obesity were treated with a restrictive diet in adulthood. Based on this, it can be assumed that KD is a safe therapy for obesity in youths, which does not cause the aggravation of kidney functioning. It has to be kept in mind that uric acid can also be considered as an important marker of metabolic syndrome and can be involved in insulin resistance, dyslipidaemia and abdominal obesity [44]. As presented and summarized in the study of Mazidi et al. [44], a lower circulating level of uric acid is associated with lower risk of diabetes, insulin resistance and better cardiovascular health. In our study, KD resulted in the lowest level of uric acid, which can be considered a positive effect. Moreover, the lower level of urea detected in rats fed with KD can be easily explained by the lack of proteins in the diet, which are the main source of urea in the circulation.

Another potential risk suggested after long-term adherence to KD is anaemia [48]. However, in the clinical trials with individuals with obesity, usually, no effect on the blood haematology and iron metabolism was reported [40,52,53]. In a clinical trial with 35 adults with obesity, no significant changes in the iron level were reported, suggesting that KD had no effect on anaemia [40]. Moreover, the clinical trial with patients treated with a KD due to drug-resistant epilepsy showed that 12-month adherence to KD resulted in an increase in HBG, HCT and MCV, while no effect on other haematological parameters (red blood cell, white blood cell and platelet counts, serum iron, total iron-binding capacity, transferrin saturation, and ferritin and folic acid levels) was observed [54]. On the contrary, the study with healthy Wistar rats showed a decrease in erythrocytes, HBG and HCT [24]. In the current investigation, similar findings were noted with an increase in HBG and HCT after KD adherence. Moreover, KD did not have a tremendous effect on the white cell and platelet systems. After restrictive KD, a reduction in WBC, LYM, and MID was reported. The observed changes in the WBC system were not varied to a high extent in any of the groups; thus, it can be assumed that no inflammatory state occurred in young rats, irrespective of the diet. However, to claim that there is no effect on the inflammation caused by KD or HFD, an additional study with analysis of cytokines has to be conducted. In addition, a restrictive KD normalized the results of the platelet system, which was elevated after the initial HFD.

Obesity is associated with chronic low-grade inflammation and, consequently, permanently increased oxidative stress [3]. The long-lasting oxidative stress damages cellular structures leading to the development of obesity-related diseases. The measurement of enzymatic antioxidant activity reflects the quality of antioxidant protection and is a commonly used marker for oxidative status measurement [55]. Fat metabolism requires more complex processes, such as reduction, oxidation, hydroxylation, and conjugation, which may elevate the production of ROS [45], therefore it can be hypothesised that KD would elevate oxidative stress. The previous studies were not conclusive about the effect of KD on oxidative stress. Arsyad et al. [24] reported that SOD decreased significantly after 60 days of KD in healthy Wistar rats. On the other hand, 3 weeks of KD in Taekwondo players were able to prevent the oxidative stress caused by exercises [56]. Nazarewicz et al. [53] did not observe any changes in the antioxidant enzymes; however, total oxidative status was improved in healthy women following KD for 2 weeks. In addition, KD was found to attenuate oxidative stress after traumatic brain injury in an animal model [23]. No effect on oxidative stress was reported in patients with obesity following very-low-calorie KD [57]. In this study, a decreasing tendency was observed for malonaldehyde, which is produced as the result of the degradation of polyunsaturated fatty acid peroxidation products and has been commonly used as a marker of oxidative stress [58]. Moreover, a clear increase in SOD, which is an enzyme of the antioxidant defence system, was noted. This together might implicate that KD does not stimulate oxidative stress, but reduces it.

Among the sources of ROS, AGEs are increasingly formed under hyperglycaemic conditions [6]. The damaging potential of AGEs have been assumed to be involved in the pathogenesis of obesity-related complications. KD have been suggested to promote the production of ROS and to be involved in protein glycation to produce N(epsilon)-(carboxyethyl)lysine, one of the AGEs [59,60]. In non-obese mice, KD resulted in a decrease in AGEs in kidneys, despite the negative changes in kidney morphology [60]. In the present study, no effect of KD on AGEs was found, suggesting that KD may be a safe option for obesity treatment in young subjects.

## 5. Conclusions

In summary, in this investigation, we demonstrated that introducing the restrictive diet in adult rats with HFD-induced obesity in adolescence resulted in a slowing down of weight gain. Our study suggests that the composition of the diet (KD vs. SD) has no effect on body weight gain/loss, but has a substantial effect on the body composition, with KD contributing to the fat mass accumulation in young animals. Increased adiposity resulted in an deterioration of liver parameters, suggesting negative changes in this organ. Therefore, KD followed as a body weight-reducing therapy should be vigilantly monitored to prevent the development of NAFLD. No adverse effects of KD were determined in haematological parameters in young rats. KD did not affect AGEs; however, a decrease in oxidative stress was observed. Based on the presented results, it can be concluded that KD, applied as a method for weight loss, may reduce oxidative stress without compromising the nutritional status; however, caution is required to control adiposity, high circulating glucose level and liver health. The efficiency of losing weight was similar, irrespective of the diet composition, and therefore the potential metabolic consequences of KD have to be carefully considered. Thus, KD applied as a weight-loss therapy should be carefully controlled, especially in young subjects.

## Figures and Tables

**Table 1:** Haematological parameters of blood before and after the intervention. Quantitative variables with a normal distribution are expressed as mean ± SD; quantitative variables which showed a non-normal distribution are expressed as median (Q1; Q3).

	Baseline		After 4 Weeks		
	SD	HFD	SD	RKD	RSD
Red blood cell system					
RBC [10^6^/µL]	7.68 ± 0.28 ^b^*	7.36 ± 0.24 ^b^	8.41 ± 0.35 ^a^	8.37 ± 0.40 ^a^	8.23 ± 0.50 ^a^
HGB [g/dL]	14.52 ± 0.57 ^a^	13.18 ± 0.65 ^c^	14.18 ± 0.33 ^a,b^	14.13 ± 0.83 ^a,b^	13.71 ± 0.81 ^b,c^
HCT [%]	42.09 ± 1.15 ^a,b^	40.59 ± 1.58 ^a,b^	41.66 ± 1.25 ^a,b^	42.11 ± 1.70 ^a^	40.29 ± 2.18 ^b^
MCV [fl]	55.00 (54.25; 55.00) ^a^	55.00 (54.25; 55.00) ^a^	49.50 (49.00; 50.75) ^b^	50.00 (49.75; 51.00) ^a,b^	49.50 (48.00; 50.00) ^b^
MCH [pg]	18.83 ± 1.00 ^a^	17.15 ± 2.03 ^b^	16.87 ± 0.58 ^b^	16.91 ± 0.57 ^b^	16.71 ± 0.28 ^b^
MCHC [g/dL]	33.95 (33.60; 34.68) ^a^	32.35 (29.30; 33.90) ^a^	34.00 (33.73; 34.20) ^a^	33.50 (33.30; 33.80) ^a^	33.90 (33.75; 34.33) ^a^
RDWc [%]	17.93 ± 0.34 ^b^	18.10 ± 0.49 ^a,b^	18.45 ± 0.31 ^a,b^	18.64 ± 0.41 ^a^	18.26 ± 0.36 ^a,b^
White blood cell system					
WBC [10^3^/µL]	6.58 ± 0.47 ^a^	6.64 ± 1.41 ^a^	6.32 ± 1.02 ^a,b^	5.43 ± 0.67 ^b,c^	4.65 ± 0.56 ^c^
LYM [10^3^/µL]	5.18 ± 0.72 ^a,b^	5.70 ± 1.24 ^a^	5.08 ± 1.04 ^a,b^	4.48 ± 0.79 ^b,c^	3.83 ± 0.38 ^c^
MID [10^3^/µL]	0.57 (0.52; 0.61) ^a^	0.20 (0.13; 0.31) ^a,b^	0.43 (0.18; 0.47) ^a^	0.16 (0.11; 0.19) ^b^	0.20 (0.12; 0.27) ^b^
GRA [10^3^/µL]	0.74 (0.72; 0.76) ^a^	0.62 (0.61; 0.63) ^a^	0.85 (0.83; 0.89) ^a^	0.79 (0.62; 0.81) ^a^	0.64 (0.50; 0.70) ^a^
LYM% [%]	82.08 (80.22; 82.48) ^a^	87.45 (83.25; 89.48) ^a^	77.90 (75.48; 82.80) ^a^	82.10 (79.88; 85.20) ^a^	85.05 (81.65; 93.00) ^a^
MID% [%]	8.39 (7.90; 9.12) ^a^	3.20 (2.23; 3.88) ^b^	7.50 (2.88; 8.38) ^a,b^	3.10 (2.00; 4.25) ^b^	4.61 (2.78; 5.30) ^a,b^
GRA% [%]	11.29 (10.85; 11.75) ^a^	9.55 (8.20; 12.85) ^a^	15.25 (13.58; 15.88) ^a^	13.85 (12.60; 14.93) ^a^	12.80 (11.13; 14.20) ^a^
Platelet system					
PLT [10^3^/µL]	613.5 ± 114.62 ^b^	769.33 ± 121.95 ^a^	687.17 ± 68.48 ^a,b^	677.75 ± 98.4 ^a,b^	724.25 ± 76.21 ^a^
PCT [%]	0.51 ± 0.10 ^b^	0.63 ± 0.12 ^a^	0.59 ± 0.06 ^a,b^	0.54 ± 0.09 ^a,b^	0.60 ± 0.07 ^a,b^
MPV [fl]	8.05 (7.80; 8.53) ^a,b^	8.20 (7.98; 8.35) ^a,b^	8.55 (8.50; 8.68) ^a^	7.90 (7.80; 8.03) ^b^	7.95 (7.78; 8.40) ^a,b^
PDWc [%]	34.48 ± 1.59 ^b^	34.85 ± 0.43 ^b^	36.75 ± 0.62 ^a^	34.99 ± 0.46 ^b^	35.11 ± 1.10 ^b^

WBC—total white blood cell; LYM—lymphocyte; MID—medium-sized cell; GRA—granulocytes; RBC—red blood cell; HGB—haemoglobin; HCT—haematocrit; MCV—mean corpuscular volume; MCH—mean corpuscular haemoglobin; MCHC—mean corpuscular haemoglobin concentration; RDWc—red cell distribution width; PLT—platelet count; PCT—platelet percentage; MPV—mean platelet volume; PDWc—platelet distribution width. (*)—different letters in superscript in the same line indicate a significant difference (*p* < 0.05) based on post hoc Fisher’s least significant difference (LSD) test.

**Table 2:** Plasma lipid profile, liver and kidney parameters in rats before and after dietary intervention. Quantitative variables with a normal distribution are expressed as mean ± SD; quantitative variables which showed a non-normal distribution are expressed as median (Q1; Q3).

	Baseline		After 4 Weeks		
	SD	HFD	SD	RKD	RSD
Lipid profile					
Cholesterol [mmol/l]	1.56 ± 0.07 ^a,b^ *	1.67 ± 0.22 ^a^	1.55 ± 0.16 ^a,b^	1.57 ± 0.16 ^a^	1.43 ± 0.13 ^b^
Tg [mmol/l]	1.01 ± 0.21 ^b^	1.34 ± 0.18 ^a^	0.95 ± 0.27 ^b,c^	1.16 ± 0.36 ^a,b^	0.70 ± 0.24 ^c^
HDL [mmol/l]	0.61 ± 0.03 ^b^	0.51 ± 0.03 ^c^	0.64 ± 0.04 ^a,b^	0.66 ± 0.05 ^a^	0.55 ± 0.07 ^c^
LDL [mmol/l]	0.95 ± 0.05 ^b^	1.17 ± 0.21 ^a^	0.91 ± 0.14 ^b^	0.91 ± 0.16 ^b^	0.87 ± 0.12 ^b^
Glucose [mmol/l]	7.38 ± 1.06 ^b^	7.28 ± 0.71 ^b^	9.18 ± 0.42 ^b^	12.73 ± 2.52 ^a^	11.89 ± 2.16 ^a^
AIP	0.21 (0.18–0.28) ^b,c^	0.42 (0.36–0.46) ^a^	0.15 (0.14–0.23) ^b,c^	0.23 (0.15–0.32) ^b^	0.08 (0.01–0.20) ^c^
Liver parameters					
AST [U/l]	67.33 ± 3.71 ^b^	70.9 ± 3.81 ^a,b^	68.37 ± 7.37 ^b^	76.3 ± 8.70 ^a^	67.26 ± 7.44 ^b^
ALT [U/l]	33.65 (31.55; 37.10) ^a,b^	46.55 (42.88; 50.00) ^a^	17.30 (15.65; 18.20) ^b^	37.70 (33.90; 41.40) ^a^	15.65 (13.48; 16.68) ^b^
ALP [U/l]	122.65 ± 14.96 ^b^	139.57 ± 29.03 ^b^	72.13 ± 11.15 ^c^	173.07 ± 27.83 ^a^	87.67 ± 19.04 ^c^
GGT [U/l]	<0.5	<0.5	<0.5	<0.5	<0.5
Bilirubin [µmol/L]	<2.9	<2.9	<2.9	<2.9	<2.9
Kidney parameters					
Uric Acid [µmol/L]	31.00 (30.00; 37.25) ^a^	23.00 (22.25; 23.75) ^b^	37.00 (33.75; 40.25) ^a^	25.00 (22.50; 27.00) ^b^	35.50 (32.25; 50.55) ^a^
Urea [mmol/l]	4.50 ± 0.65 ^a^	3.06 ± 0.32 ^c^	3.66 ± 0.76 ^b^	1.77 ± 0.29 ^d^	2.98 ± 0.41 ^c^
Creatinine [µmol/L]	18.82 ± 2.07 ^c^	18.92 ± 1.30 ^c^	22.42 ± 1.14 ^b^	23.34 ± 2.10 ^b^	25.68 ± 1.29 ^a^

Tg—triglycerides; AIP—Atherogenic Index of Plasma; ALT—alanine aminotransferase; AST—aspartate aminotransferase; ALP—alkaline phosphatase; GGT—gamma-glutamyl transferase. (*)—different letters in superscript in the same line indicate a significant difference (*p* < 0.05) based on post hoc Fisher’s least significant difference (LSD) test.

## Data Availability

Data are contained within the article or Supplementary Material.

## Supplementary Materials

The following supporting information can be downloaded at: https://www.mdpi.com/article/10.3390/nu14224805/s1, Figure S1: The correlation analysis between the analysed parameters in group fed with standard diet at the baseline; Figure S2. The correlation analysis between the analysed parameters in group fed with high-fat diet at the baseline; Figure S3. The correlation analysis between the analysed parameters in group fed with standard diet at the end of the study; Figure S4. The correlation analysis between the analysed parameters in group fed with restrictive ketogenic diet at the end of the study; Figure S5. The correlation analysis between the analysed parameters in group fed with restrictive standard diet at the end of the study.
