# Supplementary material for: The Effect of the Restrictive Ketogenic Diet on the Body Composition, Haematological and Biochemical Parameters, Oxidative Stress and Advanced Glycation End-Products in Young Wistar Rats with Diet-Induced Obesity
Source: Nutrients. 2022 Nov 13;14(22):4805. doi: 10.3390/nu14224805 (PMC9692653; doi:10.3390/nu14224805)

Figure S1. The correlation analysis between the analysed parameters in group fed with standard diet at the baseline.

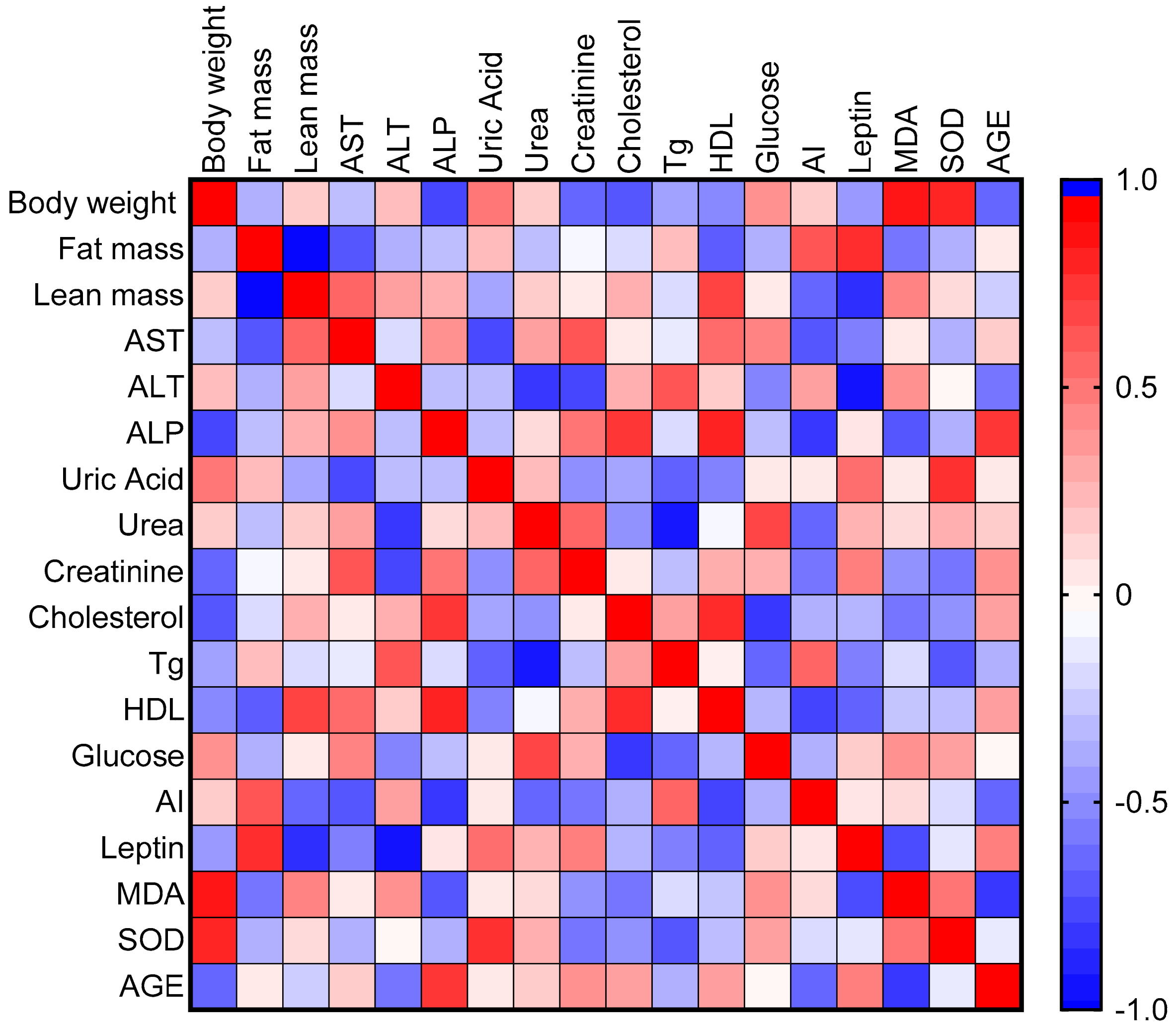

Figure S2. The correlation analysis between the analysed parameters in group fed with high-fat diet at the baseline.

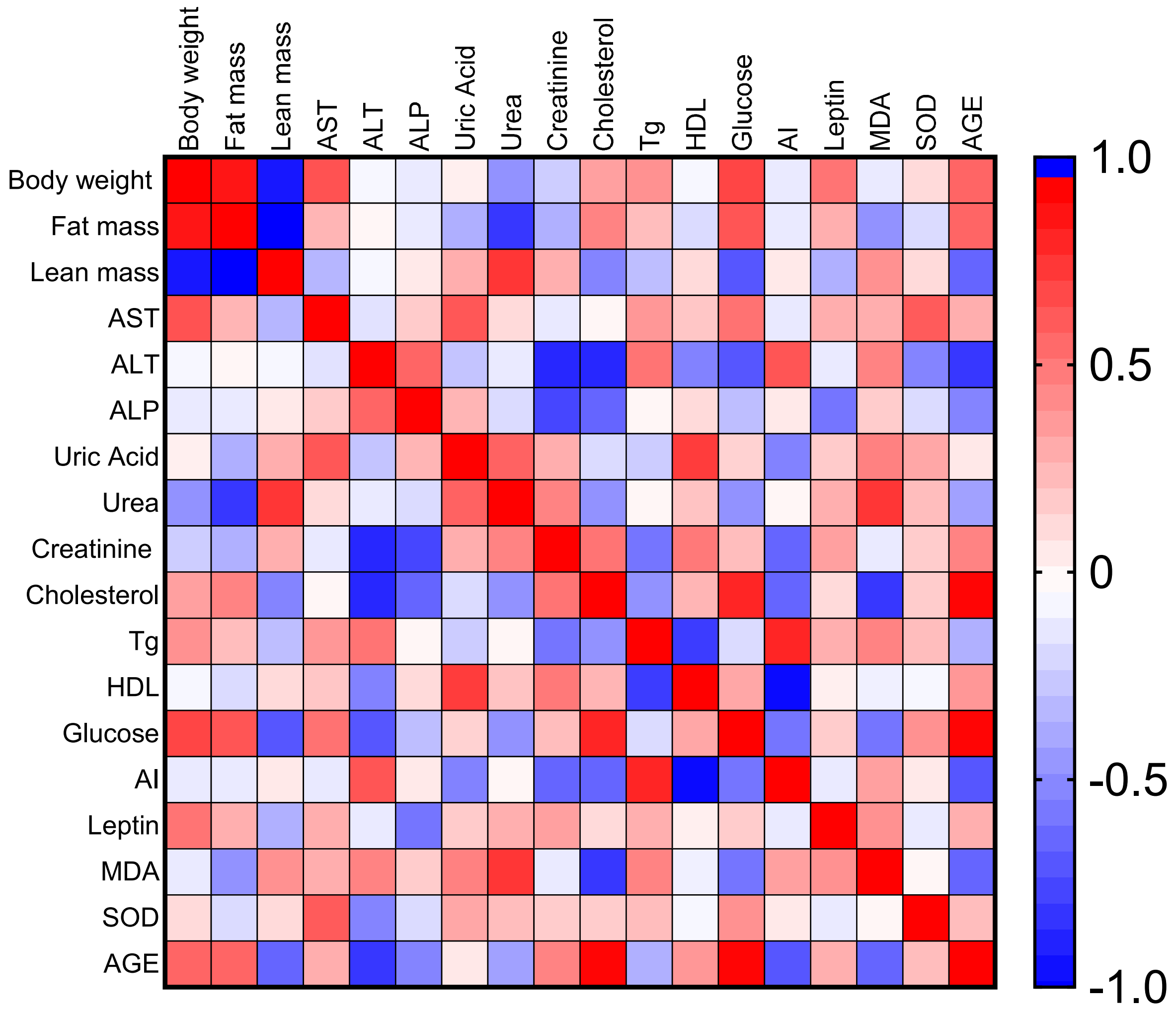

Figure S3. The correlation analysis between the analysed parameters in group fed with standard diet at the end of the study.

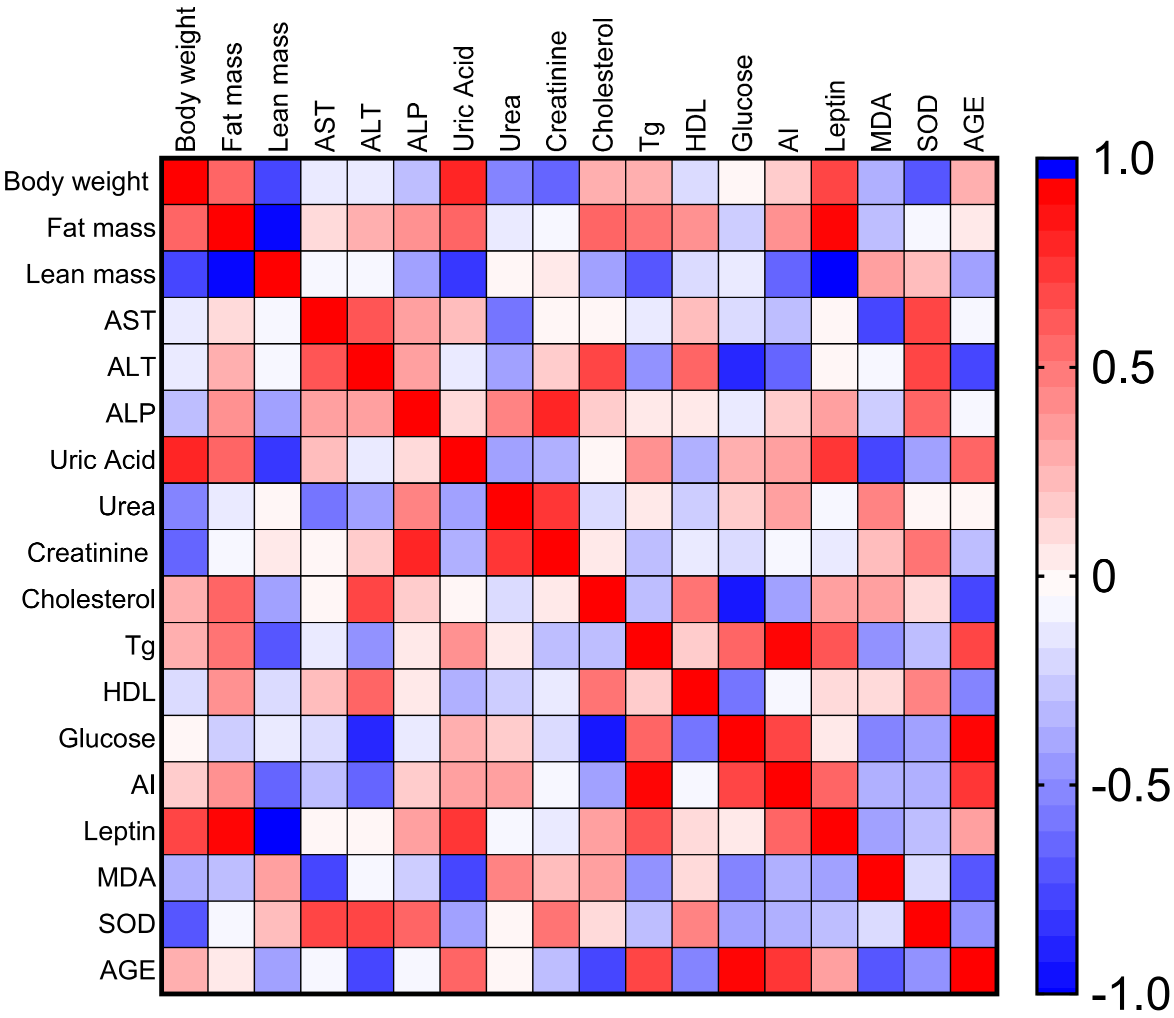

Figure S4. The correlation analysis between the analysed parameters in group fed with restrictive ketogenic diet at the end of the study.

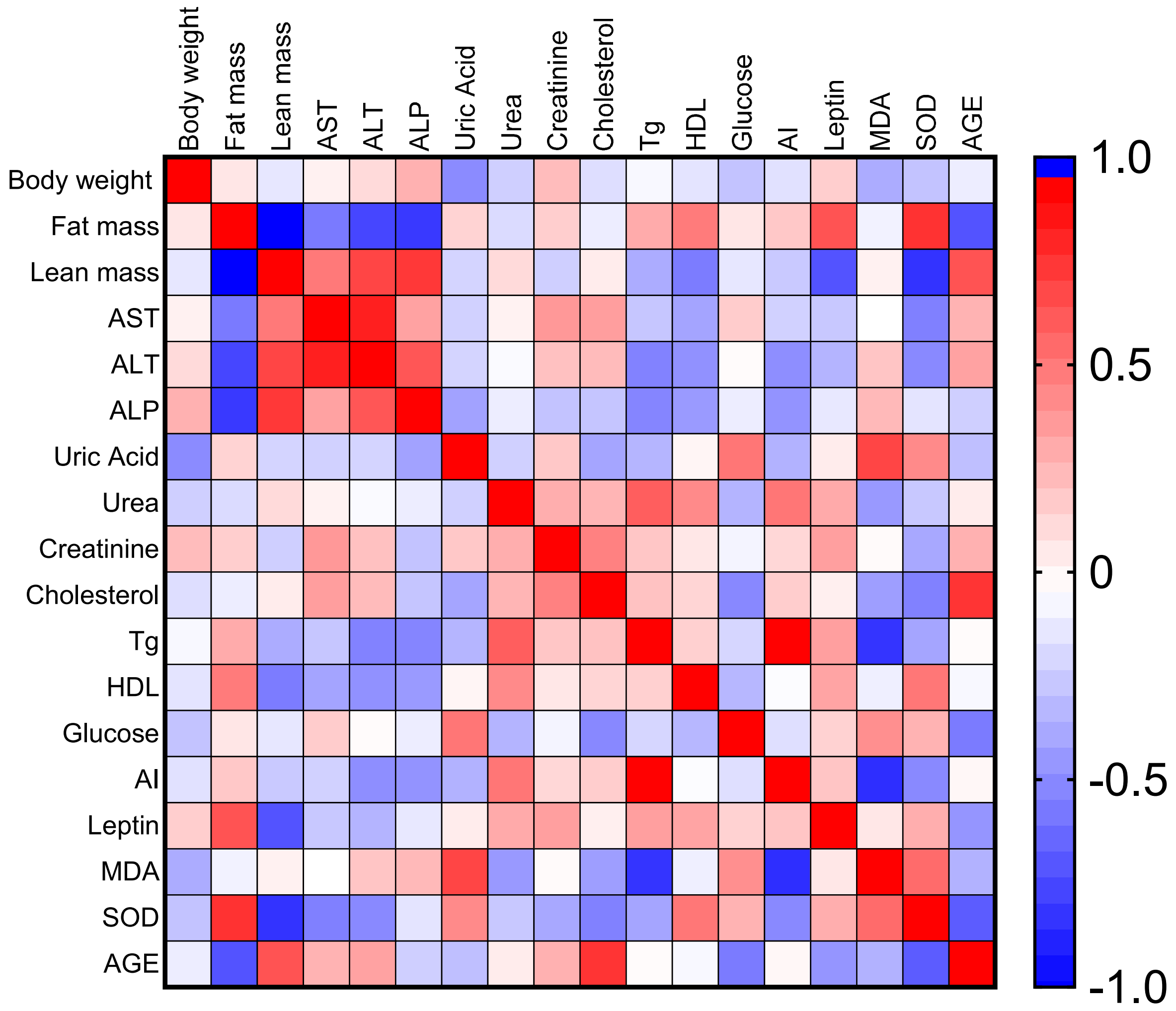

Figure S5. The correlation analysis between the analysed parameters in group fed with restrictive standard diet at the end of the study.

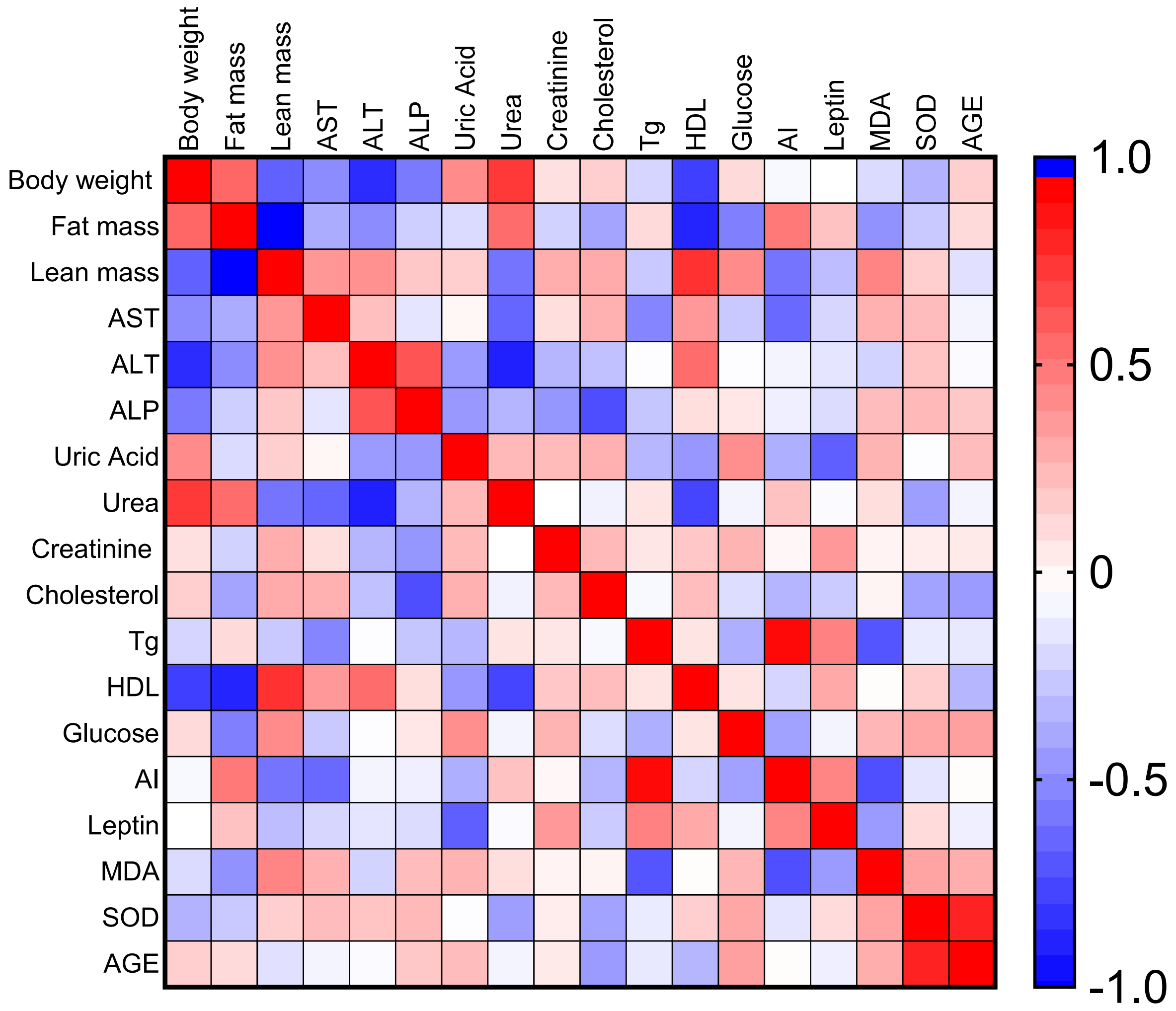

Supplement: Supplementary file 1 [file nutrients-14-04805-s001.zip › nutrients-1991286-supplementary.pdf]
